# Cannabinoid-Induced Autophagy and Heme Oxygenase-1 Determine the Fate of Adipose Tissue-Derived Mesenchymal Stem Cells under Stressful Conditions

**DOI:** 10.3390/cells9102298

**Published:** 2020-10-15

**Authors:** Katharina Bublitz, Sabine Böckmann, Kirsten Peters, Burkhard Hinz

**Affiliations:** 1 Institute of Pharmacology and Toxicology, Rostock University Medical Center, Schillingallee 70, D-18057 Rostock, Germany; katharina.bublitz@med.uni-rostock.de (K.B.); sabine.boeckmann@med.uni-rostock.de (S.B.); 2 Department of Cell Biology, Rostock University Medical Center, Schillingallee 69, D-18057 Rostock, Germany; kirsten.peters@med.uni-rostock.de

**Keywords:** cannabinoids, heme oxygenase-1, adipose tissue-derived mesenchymal stem cells, autophagy

## Abstract

The administration of adipose tissue-derived mesenchymal stem cells (ADMSCs) represents a promising therapeutic option after myocardial ischemia or myocardial infarction. However, their potential is reduced due to the high post-transplant cell mortality probably caused by oxidative stress and mitogen-deficient microenvironments. To identify protection strategies for ADMSCs, this study investigated the influence of the non-psychoactive phytocannabinoid cannabidiol (CBD) and the endocannabinoid analogue R(+)-methanandamide (MA) on the induction of heme oxygenase-1 (HO-1) and autophagy under serum-free conditions. At a concentration of 3 µM, CBD induced an upregulation of HO-1 mRNA and protein within 6 h, whereas for MA only a late and comparatively lower increase in the HO-1 protein could be detected after 48 h. In addition, both cannabinoids induced time- and concentration-dependent increases in LC3A/B-II protein, a marker of autophagy, and in metabolic activity. A participation of several cannabinoid-binding receptors in the effect on metabolic activity and HO-1 was excluded. Similarly, knockdown of HO-1 by siRNA or inhibition of HO-1 activity by tin protoporphyrin IX (SnPPIX) had no effect on CBD-induced autophagy and metabolic activity. On the other hand, the inhibition of autophagy by bafilomycin A_1_ led to a significant decrease in cannabinoid-induced metabolic activity and to an increase in apoptosis. Under these circumstances, a significant induction of HO-1 expression after 24 h could also be demonstrated for MA. Remarkably, inhibition of HO-1 by SnPPIX under conditions of autophagy deficit led to a significant reversal of apoptosis in cannabinoid-treated cells. In conclusion, the investigated cannabinoids increase metabolic viability of ADMSCs under serum-free conditions by inducing HO-1-independent autophagy but contribute to apoptosis under conditions of additional autophagy deficit via an HO-1-dependent pathway.

## 1. Introduction

Mesenchymal stem cells (MSCs) from human adipose tissue were first isolated in 2001 and have since been referred to as adipose tissue-derived MSCs (ADMSCs) [1]. They represent an alternative source of autologous adult stem cells, which are usually available in large quantities and can be cultivated in vitro for extended periods with stable population doubling and low levels of senescence [1]. ADMSCs are, therefore, considered an ideal source for tissue engineering and have a high application and research value. Similar to bone marrow stem cells, ADMSCs secrete many different types of growth factors and cytokines that can contribute to the stimulation of angiogenesis and myocardial regeneration after ischemic injury [2,3]. Angiogenesis, a biological process in which existing blood vessels form new blood vessels, is an important therapeutic goal in the treatment of ischemic heart disease [4,5]. However, the high cell mortality of ADMSCs after transplantation, probably caused by oxidative stress induced by ischemia and mitogen-deficient microenvironments, may limit their potential to improve heart repair [6].

The discovery of the endocannabinoid system with the integrated components CB_1_ and CB_2_ receptors, the endocannabinoid ligands anandamide and 2-arachidonoylglycerol as well as the enzymes involved in their synthesis and degradation has led to a strong interest in the physiological and pathophysiological effects influenced thereby as well as in the pharmacological modulation of this system (for review see [7,8]). In this context, studies in recent years have demonstrated a protective effect of cannabinoids on the survival of cardiovascular cells under ischemic and nutrient-poor conditions [9,10]. Further investigations have shown that MSCs from adipose tissue as well as MSCs from other sources express the complete endocannabinoid system [11,12,13,14]. The endocannabinoids as well as the phytocannabinoids cannabidiol (CBD) and Δ^9^-tetrahydrocannabinol (THC) stimulate the migration of ADMSCs, which is considered an important function in wound healing and tissue regeneration [12,13,14].

The cytoprotective microsomal heme oxygenase (HO) system consists of the inducible HO-1 and the constitutive HO-2 isoenzyme, which catalyze the first rate-limiting step of the degradation of heme into the antioxidative reaction products biliverdin and bilirubin [15,16]. In particular, the isoenzyme HO-1 plays an important role in cell survival under oxidative stress conditions [15,16]. For example, overexpression of HO-1 or pretreatment with various HO-1 inducers was associated with a better survival of ADMSCs against cellular stress and various oxidative stimuli [17,18,19]. Furthermore, it could be shown that supernatants obtained from HO-1-transfected MSCs led to improved left ventricular function, limited myocardial infarction size, and inhibition of cardiomyocyte apoptosis in a model of rat myocardial infarction [20].

According to studies in recent years, HO-1 appears to interact with other stress systems. In fact, HO-1-dependent autophagy has been described in various cells [21,22,23]. Autophagy, a process of cellular self-digestion, is considered to be a cytoprotective response that may occur after the withdrawal of growth factors or under stress conditions (for review see [24]). In this context, autophagy has also been identified as an essential mechanism for protecting MSCs and regulating bioenergetic requirements for stem cell activation [25,26,27,28]. On the other hand, initial data from experiments with ADMSCs have also shown that the downregulation of nuclear factor erythroid 2-related factor 2 (Nrf2)/HO-1 promotes autophagy-dependent osteoblastic differentiation, which indicates a negative regulation of autophagy by HO-1 in this cell type [29].

In the present study, the influence of two cannabinoids, the non-psychoactive phytocannabinoid cannabidiol (CBD) and the endocannabinoid analogue R(+)-methanandamide (MA), was investigated with regard to the regulation of HO-1 induction, autophagy, and metabolic activity of ADMSCs. Here we show that the cannabinoids examined increase autophagy and metabolic activity of ADMSCs under serum-free conditions independent of HO-1 induction but contribute to apoptosis under conditions of additional autophagy inhibition in an HO-1-dependent manner.

## 2. Materials and Methods

### 2.1. Materials

CBD was supplied by Biotrend (Cologne, Germany) and MA as well as O-1602 by TOCRIS (Bristol, UK). AM-251 and AM-630 were bought from Biomol GmbH (Hamburg, Germany). Capsazepine was obtained from Sigma-Aldrich (Taufkirchen, Germany). Bafilomycin A_1_ was obtained from InvivoGen (Toulouse, France). HO-1 antibody (ADI-SPA-895), HO-2 antibody (ADI-SPA-897), and tin protoporphyrin IX (SnPPIX) were obtained from Enzo Life Sciences GmbH (Lörrach, Germany). Cleaved caspase-3 (Asp175) (5A1E) antibody (#9664), LC3A/B antibody (#4108), and secondary antibodies (anti-rabbit antibody, #7074; anti-mouse antibody, #7076) were purchased from Cell Signaling Technology Europe (Frankfurt/Main, Germany). β-Actin antibody (clone AC-74, #A5316) was obtained from Sigma-Aldrich (Taufkirchen, Germany). siRNA targeting HO-1 was purchased from Santa Cruz Biotechnology, Inc. (Heidelberg, Germany; sc-35554). Negative control siRNA (cat. no. 1022076) was from Qiagen (Hilden, Germany). Transfection reagent Lipofectamine^TM^ RNAiMAX and transfection medium Opti-MEM^®^ I Reduced Serum Medium were obtained from Thermo Fisher Scientific Inc. (Schwerte, Germany). Dulbecco’s Modified Eagle’s medium (DMEM, high glucose, GlutaMAX™), penicillin-streptomycin, and trypsin-EDTA were purchased from Gibco^®^ from Life Technologies™ (Darmstadt, Germany). Fetal bovine serum (fetal calf serum, FCS) was from PAN™ Biotech GmbH (Aidenbach, Germany).

### 2.2. Cell Culture

Subcutaneous adipose tissue was obtained by liposuction. The tissue donation was approved by the Ethics Committee of the Rostock University Medical Center. It complies with the ethical standards of the World Medical Association Declaration of Helsinki. Informed consent was obtained from all patients for the creation of cell models from their tissue. The adipose tissue was digested in a collagenase NB4 standard solution (6 mg/mL, SERVA, Heidelberg, Germany) while gently stirring for 30 min at 37 °C. The digested adipose tissue was then filtered through 100 µm nylon tissue (BD Falcon™ Cell Strainer, BD Biosciences, Heidelberg, Germany) to separate the adipocyte fraction from the stromal fraction. The stromal fraction was then washed in PBS with 10% FCS and sedimented. The lower part of the filtrate was then filtered through a 40 µm nylon mesh (BD Falcon^TM^ Cell Strainer, BD Biosciences, Heidelberg, Germany). Both fractions were centrifuged at 400× *g* for 10 min. The supernatant in both fractions was removed and the pellets were combined, resuspended in PBS with 10% FCS, and centrifuged at 400× *g* for 5 min. All centrifugation steps were performed at room temperature. The cells were cultivated in DMEM with 10% FCS and 100 U/mL penicillin and 100 µg/mL streptomycin and grown in a humidified incubator at 37 °C and 5% CO_2_. After about 24 h, the ADMSCs were separated from the other adherent cells of the primary culture by their characteristic expression of the CD34 surface antigen. The Dynabeads^®^ CD34-positive isolation kit (Invitrogen, Karlsruhe, Germany) was used according to the manufacturer’s instructions.

Experiments were performed with cells from passage 4, either freshly isolated ADMSCs or thawed. Cryopreservation of the ADMSCs was always performed in passage 2. The cells were washed with PBS, trypsinated, and centrifuged as usual. An amount of 350 µL FCS and 150 µL DMSO were added to 1 mL cell suspension and transferred to cryovials. The cryovials were stored at −80 °C until the next day at the earliest.

The ADMSCs were seeded at a density of 2 × 10^4^ cells per cm^2^. Since each experiment was performed on a 6-well plate, 192,000 cells per well were always seeded. All incubations were performed in serum-free DMEM containing 100 U/mL penicillin and 100 µg/mL streptomycin.

siRNAs were dissolved in RNase-free water according to the manufacturer’s instructions. Test substances were dissolved in ethanol, DMSO, or NaOH, with the corresponding solvents showing final concentrations in the incubates of 0.1% (*v*/*v*) ethanol (for CBD and MA), 0.1% (*v*/*v*) DMSO (for AM-251, AM-630, capsazepine, O-1602 and bafilomycin A_1_), or 0.001 M NaOH (for SnPPIX). The respective vehicle control incubate contained the corresponding concentration of ethanol, DMSO, or NaOH of the test substance incubates.

### 2.3. Cell Metabolic Activity Assay

The metabolic activity (metabolic viability) of the cells was determined with the colorimetric WST-1 test (Sigma-Aldrich, Taufkirchen, Germany). This assay is based on the cleavage of WST-1, a water-soluble tetrazolium salt (4-[3-(4-iodophenyl)-2-(4-nitrophenyl)-2H-5-tetrazolio]-1,3-benzene disulfonate), to a soluble formazan dye, whereby this bioreduction depends largely on the glycolytic production of NAD(P)H in viable cells. In this respect, the amount of formazan dye formed correlates directly with the number of metabolically active cells in the culture. The cells were seeded in a 6-well plate and allowed to grow to confluence before being exposed to serum-free DMEM and immediately treated with test substances or vehicles for the specified times. After the incubation period, the medium was renewed and the WST-1 reagent was added to cells yielding a final dilution of 1:10. Cell metabolic activity was determined by measuring the absorbance at 450/690 nm with an ELISA plate reader. In the case of subsequent protein analysis, the cells were then lysed and further examined using Western blot.

### 2.4. Trypan Blue Staining

To assess whether the increased metabolic activity was due to increased metabolism or proliferation, the cell count was determined by trypan blue staining. Since trypan blue cannot pass the membrane of intact cells, only dead cells are stained due to their permeable membrane. In this assay, cells were seeded in a 6-well plate as described above and trypsinized after a 24-h incubation with the test substances or vehicle. The resulting cell pellet was transferred to fresh DMEM. The analysis was performed on samples diluted 1:2 (*v*/*v*) with 0.4% trypan blue (Gibco^®^ from Life Technologies™) in an automated cell counter Luna-II™ (Biozym Scientific GmbH, Hess. Oldendorf, Germany), followed by lysis of the remaining cell pellet and analysis of the proteins by Western blot.

### 2.5. siRNA Transfection

Knockdown of HO-1 protein expression was performed with the Lipofectamine™ RNAiMAX transfection reagent in reverse procedure according to the instructions of the manufacturer (Invitrogen, Karlsruhe, Germany). Transfection complexes were generated by mixing 3 µL of a 10 µM HO-1 specific siRNA or 1.5 µL of a 20 µM non-target siRNA stock solution with 5 µL Lipofectamine™ RNAiMAX in Opti-MEM^®^ I Reduced Serum Medium in a total volume of 500 µL, this is the approach for a well. The complexes were thoroughly mixed and incubated for about 20 min at room temperature. An amount of 2 mL of cell suspension were seeded in a 6-well plate, as described above, after 500 µL transfection complex was added. The control cells were transfected in parallel with non-silencing siRNA to demonstrate specific gene silencing. The final concentration of siRNA was 12 nM. After 24 h, the supernatant was aspirated and the cells were incubated in serum-free medium, as described above, for 24 h. Subsequently, the metabolic activity was determined with the WST-1 reagent and the adherent cells were harvested for Western blot analysis.

### 2.6. Quantitative RT-PCR Analysis

For quantification of HO-1 und HO-2 mRNA expression, ADMSCs were seeded in 6-well plates and treated as described. After stimulation and incubation of the test substances or vehicle for the indicated time, total RNA was isolated using RNeasy Mini Kit from Qiagen. HO-1, HO-2, and β-Actin mRNA levels were determined by real-time RT-PCR using the Applied Biosystems^®^ TaqMan^®^ RNA-to-CT™ 1-Step Kit (Thermo Fisher Scientific Inc.). Primers and probes for human β-actin, HO-1, and HO-2 were Applied Biosystems^®^ TaqMan^®^ Gene Expression Assay products (Thermo Fisher Scientific Inc.). All experiments were performed according to the manufacturer’s instructions. HO-1 and HO-2 mRNA levels were normalized to β-actin.

### 2.7. Western Blot Analysis

For the analysis of HO-1, HO-2, LC3A/B-I/II, and β-actin protein values, ADMSCs seeded in 6-well plates were lysed after treatment with the test substances or their vehicles. Only the adherent cells were lysed in the solubilization buffer (50 mM HEPES (pH 7.4), 150 mM NaCl, 1 mM EDTA, 1% (*v*/*v*) Triton^®^ X-100, 10% (*v*/*v*) glycerol, 1 mM PMSF, 1 µg/mL leupeptin, 0.5 mM orthovanadate, and 10 µg/mL aprotinin) after the specified incubation time, followed by homogenization, shaking on ice for 30 min and centrifugation at 20,817× *g* for 5 min.

For the analysis of cleaved caspase-3 protein levels, a different method of protein processing was also used for HO-1 and LC3A/B-I/II in the corresponding experiments (see later Figures 5–8). Here, after incubation with the test substances or their vehicles, cell-culture media (non-adherent cells), and trypsinated (adherent) cells were collected per well for the specified times and centrifuged at 500× *g*. Each cell pellet was lysed in 50 µL sample buffer (62.5 mM Tris-HCl, 2% (*v*/*v*) SDS, 10% (*v*/*v*) glycerol), boiled at 95 °C for 5 min, homogenized by sonication and centrifuged at 13,800× *g* for 3 min.

In the case of both described methods of protein extraction, the total protein concentration in the supernatants obtained after the final centrifugation step was determined with the Pierce™ bicinchoninic acid (BCA) protein assay kit (Thermo Fisher Scientific Inc.) according to the manufacturer’s protocol.

The proteins were separated on a 12% sodium dodecyl sulfate polyacrylamide gel. After transfer to nitrocellulose and blocking of the membranes with 5% milk powder the blots were examined with specific primary antibodies. To detect the respective proteins, the membranes were probed with horseradish-peroxidase-conjugated rabbit or mouse secondary antibodies. Antibody binding was visualized by a chemiluminescent solution (100 mM Tris-HCl (pH 8.5), 1.25 mM luminol, 200 µM p-coumaric acid, 0.09% (*v*/*v*) hydrogen peroxide, 0.0072% (*v*/*v*) DMSO). The densitometric analysis of the band intensities was achieved by optical scanning and quantification with the 1-D analysis software Quantity One (Bio-Rad, Munich, Germany). After the analysis, the membranes were stripped and reprocessed. To evaluate changes in protein expression, the vehicle controls were defined as 100%. To show a uniform protein load, the membranes were examined with an antibody against β-actin. All densitometric values (HO-1, HO-2, LC3A/B-I, LC3A/B-II, caspase-3) were normalized to those of β-actin. The LC3A/B-II/I ratios were accordingly calculated from the β-actin-normalized LC3A/B-II and LC3A/B-I values.

### 2.8. Statistics

All values are presented as means ± standard error of the mean (SEM). The n numbers given in the figure and the table legends refer to the number of donors examined. In the case of the WST-1 tests, an individual donor value represents the average of 3 to 4 measurements on the cells of one donor in order to achieve a higher measurement accuracy. However, only the average values of the individual donors were included in the presentation and statistical evaluations here. Comparisons between 2 groups were carried out using Student’s unpaired *t* test. Comparisons between more than 2 groups were performed by one-way ANOVA with Bonferroni´s or Dunnett´s post hoc test. In the case of Bonferroni’s post hoc test, the determination of statistical significance was limited to the groups of interest for reasons of clarity of presentation. All statistical analyses were conducted with GraphPad Prism 5.0 (GraphPad Software, Inc., San Diego, CA, USA).

## 3. Results

### 3.1. CBD Induces HO-1 mRNA and Protein Expression in ADMSCs under Serum-Free Conditions

**Figure 1:**
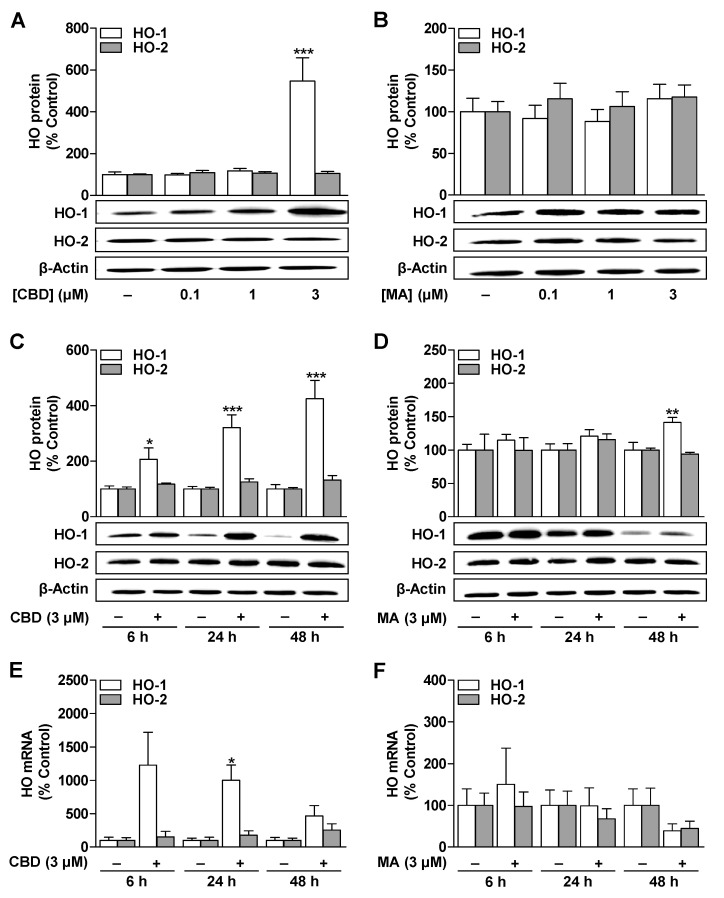
Impact of CBD and MA on the expression of HO-1 and HO-2 protein and mRNA in ADMSCs. Concentration-dependent effect of CBD (**A**) and MA (**B**) on the expression of HO-1 and HO-2 protein. The cells were incubated for 24 h with the indicated concentration of CBD and MA or with vehicle. Time-course of expression of HO-1 and HO-2 protein (**C**,**D**) and mRNA (**E**,**F**) in ADMSCs treated with CBD (3 µM), MA (3 µM), or vehicle. Protein and mRNA expression values were normalized to β-actin. The percentage control represents a comparison with the cells treated with vehicle (set to 100%) in the absence of the test substance. The data represent mean values ± SEM of *n* = 9 ((**A**), HO-1), *n* = 8 ((**A**), HO-2), *n* = 7 ((**B**), HO-1), *n* = 4 ((**B**), HO-2), *n* = 8 ((**C**), HO-1), *n* = 7 ((**C**), HO-2), *n* = 8 ((**D**), HO-1), *n* = 4 ((**D**), HO-2), or *n* = 3 (**E**,**F**) different donors. * *p* < 0.05, ** *p* < 0.01, *** *p* < 0.001 vs. time-matched vehicle control; one-way ANOVA with Dunnett’s post hoc test (**A**,**B**) or Student’s two tailed *t* test (**C**–**F**).

To determine whether CBD and MA increase HO-1 or HO-2 expression in ADMSCs under serum-free conditions, the cells were treated with the substances for 6 to 48 h. A 24-h incubation of cells with CBD at concentrations of 0.1 to 3 µM resulted in a significant increase in the expression of the HO-1 protein when using the 3 µM concentration (Figure 1A). In contrast, MA did not induce the HO-1 protein at any concentration tested at this time (Figure 1B). The induction of the HO-1 protein and HO-1 mRNA by CBD was time-dependent (Figure 1C,E) and, in the case of the protein, led to maximum stimulation after a 48-h incubation period (Figure 1C). On the other hand, a comparatively lower increase in the HO-1 protein for MA could only be measured after a 48-h incubation period (Figure 1D), which, however, could not be determined at the mRNA level (Figure 1F).

### 3.2. CBD and MA Increase Metabolic Activity and Autophagy of ADMSCs under Serum-Free Conditions

To determine whether CBD or MA affect metabolic activity (metabolic viability) and autophagy of ADMSCs, cells were incubated with the respective substance in concentrations of 0.1 to 3 µM for 24 h and subsequently subjected to WST-1 assays and Western blot analysis.

**Figure 2:**
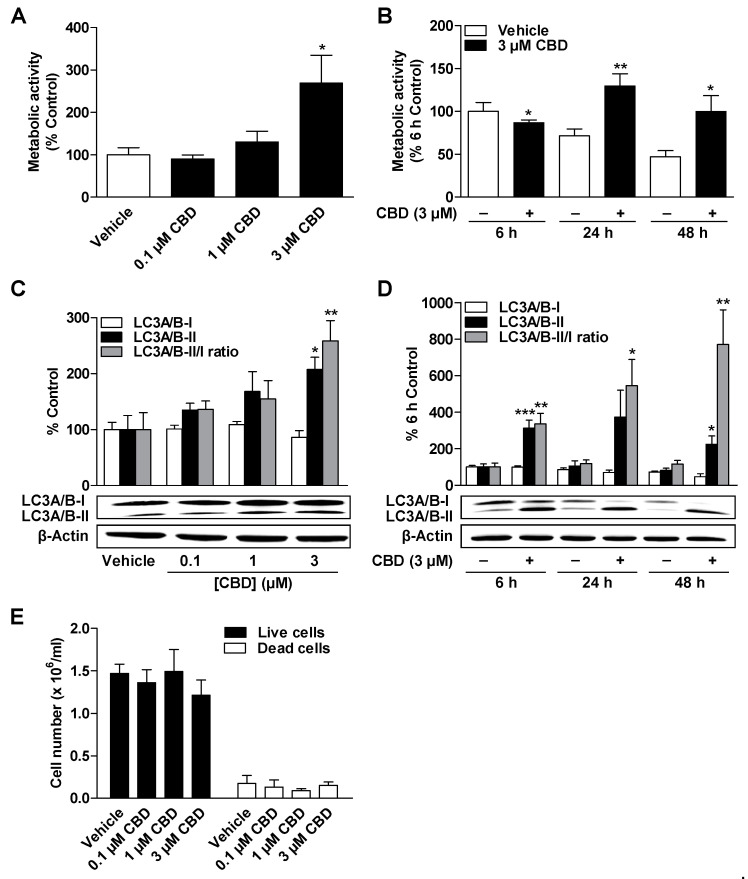
Concentration- and time-dependent effect of CBD on metabolic activity, autophagy, and cell count of ADMSCs. The cells were incubated with the indicated concentration of CBD or vehicle for 24 h (**A**,**C**,**E**) or with 3 µM CBD or vehicle for the specified times (**B**,**D**). After incubation, the cells were analyzed for their metabolic activity by WST-1 assay (**A**,**B**), for their LC3A/B-I/II protein expression by Western blot analysis (**C**,**D**), and for their cell count (**E**) by trypan blue staining. The protein expression values were normalized to β-actin. The percentage control represents a comparison with the time-matched vehicle-treated cells (**A,C**) or the cells treated with vehicle for 6 h (**B**,**D**). The data represent mean values ± SEM of *n* = 6–8 (**A**), *n* = 5 (**C**), *n* = 7 (**B**,**D**), or *n* = 3 (**E**) different donors. * *p* < 0.05, ** *p* < 0.01, *** *p* < 0.001 vs. time-matched vehicle; one-way ANOVA with Dunnett’s post hoc test (**A**,**C**) or Student´s *t* test (**B**,**D**).

**Figure 3:**
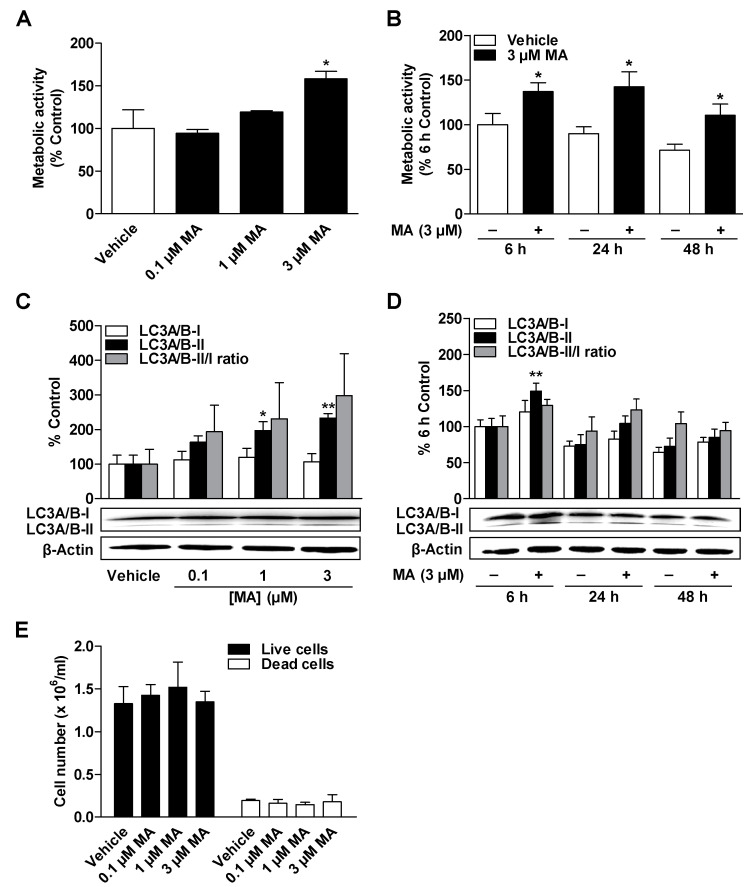
Concentration- and time-dependent effect of MA on metabolic activity, autophagy, and cell count of ADMSCs. The cells were incubated with the indicated concentration of MA or vehicle for 24 h (**A**,**C**,**E**) or with 3 µM MA or vehicle for the specified times (**B**,**D**). After incubation, the cells were analyzed for their metabolic activity by WST-1 assay (**A**,**B**), for their LC3A/B-I/II protein expression by Western blot analysis (**C**,**D**), and for their cell count (**E**) by trypan blue staining. The protein expression values were normalized to β-actin. The percentage control represents a comparison with the time-matched vehicle-treated cells (**A**,**C**) or the cells treated with vehicle for 6 h (**B**,**D**). The data represent mean values ± SEM of n = 4 (**A**,**C**), *n* = 6 (**B**), *n* = 8 (**D**), or *n* = 3 (**E**) different donors. * *p* < 0.05, ** *p* < 0.01 vs. time-matched vehicle; one-way ANOVA with Dunnett’s post hoc test (**A**,**C**) or Student’s *t* test (**B**,**D**).

As shown in Figure 2A and Figure 3A, the metabolic activity of ADMSCs was increased after a 24-h incubation period with CBD and MA at concentrations of 1 to 3 µM, respectively, which was significant in the case of the 3 µM concentrations of both cannabinoids. CBD and MA increased the metabolic activity time dependently compared to time-matched vehicle control with the comparatively greatest effects after a 24-h incubation period (Figure 2B and Figure 3B). To show the time-dependent decrease in metabolic activity in the vehicle-treated cells, all values were normalized to the 6-h vehicle control.

According to Figure 2C and Figure 3C, CBD and MA induced autophagy depending on concentration, as demonstrated by a significant increase in the autophagy marker LC3A/B-II. The increased expression of the LC3A/B-II protein by both cannabinoids at a concentration of 3 µM each was time-dependent over a period of 6 to 48 h (Figure 2D and Figure 3D) but led to comparatively stronger stimulation by CBD. The LC3A/B-II/I ratio also showed a concentration- and time-dependent increase for both cannabinoids, which was significant for CBD-treated cells. Under the same experimental conditions, no change in the number of living and dead cells could be recorded in the presence of both cannabinoids (Figure 2E and Figure 3E).

### 3.3. Cannabinoid-Binding Receptors Mediate neither the CBD-Increased HO-1 Expression nor the CBD- and MA-Increased Metabolic Activity of ADMSCs

Next, a possible role of the cannabinoid-binding receptors CB_1_, CB_2_, TRPV1, and (in the case of CBD) GPR55 as potential mediators of the metabolic activity-promoting and HO-1-inducing effects of both cannabinoids was investigated. For this purpose, the cells were preincubated with the CB_1_ receptor antagonist AM-251, the CB_2_ receptor antagonist AM-630, the TRPV1 antagonist capsazepine, and the GPR55 agonist O-1602. All antagonists were used at a concentration of 1 μM, which in previous studies led to interferences with CB_1_-, CB_2_- and TRPV1-dependent cannabinoid effects [12,13,14,30,31,32]. The same applies to the GPR55 agonist O-1602, which, at a concentration of 1 µM, has been shown to inhibit the action of the GPR55 antagonist CBD in ADMSCs [12]. The results of this approach showed that neither the receptor antagonists nor O-1602 caused a significant change in the increases in metabolic activity and HO-1 induction caused by both cannabinoids (Table 1 and Table 2), suggesting receptor-independent events as the underlying mechanism. Furthermore, neither the antagonists nor O-1602 per se had a significant effect on the basal HO-1 expression (Table 1 and Table 2).

### 3.4. HO-1 Does not Mediate the CBD-Induced Increase in Metabolic Activity and Autophagy of ADMSCs

**Figure 4:**
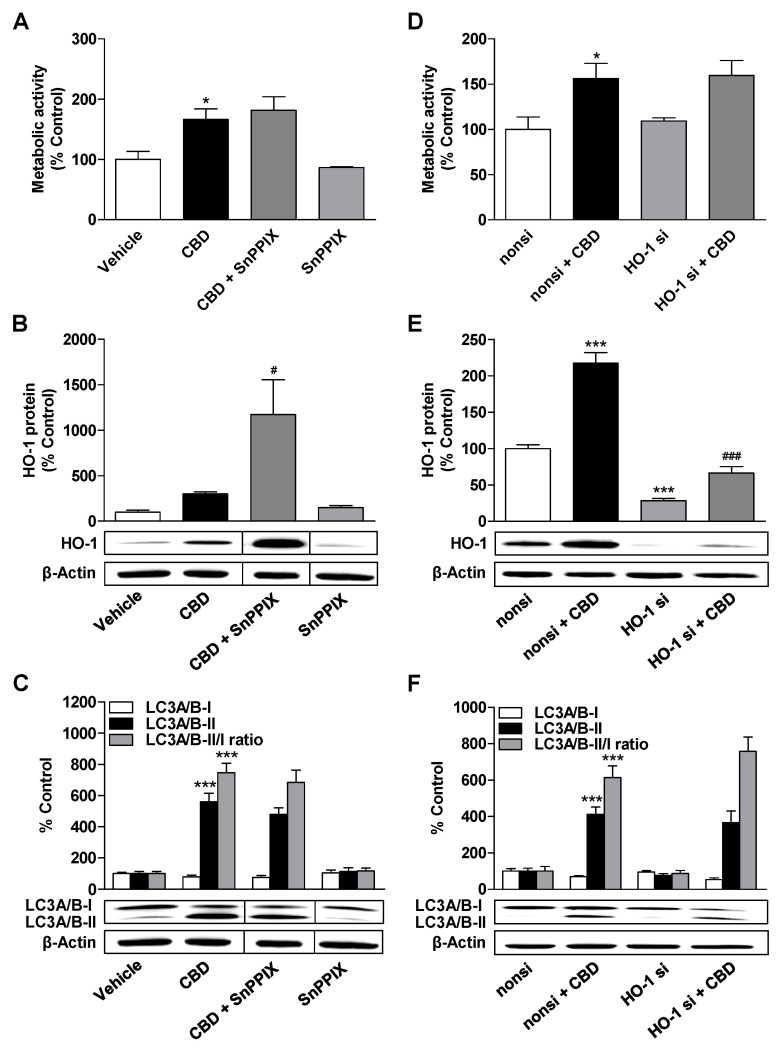
Impact of the HO-1 inhibitor SnPPIX and HO-1 siRNA on the effects of CBD on metabolic activity, autophagy, and HO-1 expression. ADMSCs were pretreated with the HO-1 inhibitor SnPPIX (25 µM) for 30 min prior to CBD (3 µM) stimulation for 24 h (**A**–**C**). In other experiments (**D**–**F**), the cells were transfected with HO-1 specific siRNA (HO-1 si) or nonsilencing siRNA (nonsi; negative control siRNA) at a final concentration of 12 nM and then incubated with CBD (3 µM) or vehicle for another 24 h. After the respective incubation periods, the cells were analyzed for their metabolic activity (**A**,**D**) and for the expression of HO-1 (**B**,**E**) and LC3A/B-I/II (**C**,**F**) protein. The protein expression values were normalized to β-actin. The vertical black lines inside the boxes of some blots (**B**,**C**) indicate that the lanes in between have been removed, so here too, signals from protein samples loaded onto the same gel were compared. The same actin blot was used as control in (**B**,**C**), since the proteins analyzed in these Western blots were resolved on the same gel. The percentage control represents a comparison with the group treated with the vehicle (set to 100%). The data represent mean values ± SEM of n = 6 different donors (**A**–**F**). * *p* < 0.05, *** *p* < 0.001 vs. vehicle or nonsilencing siRNA control; # *p* < 0.05, ### *p* < 0.001 vs. CBD (**B**) or nonsi + CBD (**E**); one-way ANOVA with Bonferroni’s post hoc test.

To clarify a possible relationship between the induction of HO-1 by CBD and its metabolic activity and autophagy enhancing effect shown above, we investigated whether the HO inhibitor SnPPIX reduces the latter two effects of CBD (Figure 4).

As shown in Figure 4A,C, SnPPIX did not significantly inhibit CBD-mediated increases in metabolic activity, LC3A/B-II protein expression, and LC3A/B-II/I ratio. Thus, HO-1 induction obviously does not play an essential role in these processes. Interestingly, these effects of the HO-1 inhibitor SnPPIX could be registered despite increased HO-1 protein expression occurring during combined incubation of CBD with SnPPIX (Figure 4B).

To confirm the results with SnPPIX and to exclude possible off-target effects of this HO-1 inhibitor, experiments with selective HO-1 siRNA were performed. Relative to cells transfected with a negative control siRNA, HO-1 siRNA completely reduced the upregulation of HO-1 expression by 3 µM CBD (Figure 4E). However, no significant effect on CBD-mediated increases in metabolic activity, LC3A/B-II protein expression, and LC3A/B-II/I ratio could be observed under conditions of HO-1 downregulation (Figure 4D,F). Thus, these experiments also support the conclusion that HO-1 is not involved in the modulation of metabolic activity and autophagy by CBD.

### 3.5. Inhibition of Autophagy Leads to a Decrease in the Metabolic Activity Elevated by CBD and MA, to an Increase in Caspase-3 Cleavage, and to the Induction of HO-1 Expression in MA-Treated ADMSCs

**Figure 5:**
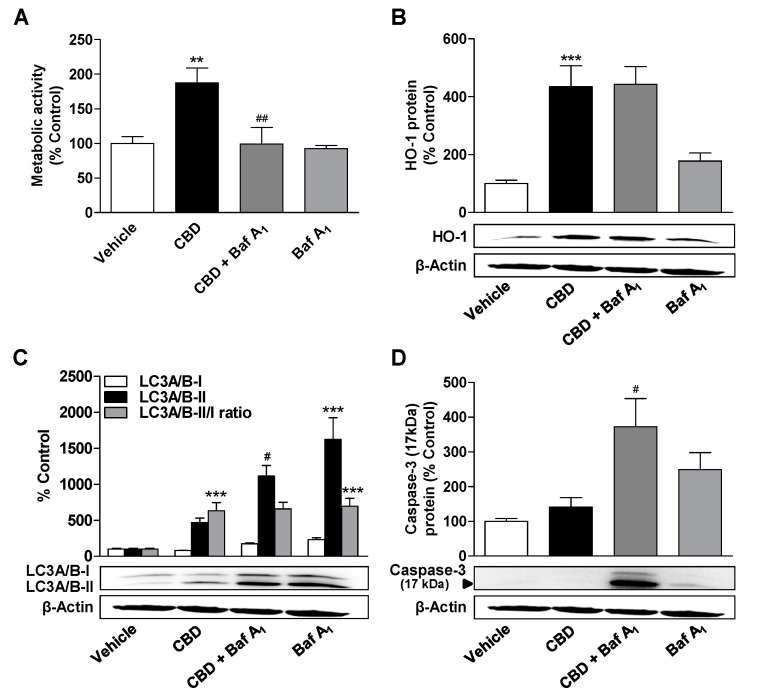
Influence of the autophagy inhibitor bafilomycin A_1_ (Baf A_1_) on the effects of CBD on metabolic activity, autophagy, apoptosis, and HO-1 expression. ADMSCs were pretreated for 30 min with bafilomycin A_1_ (100 nM) and after subsequent addition of CBD (3 µM) or vehicle, the cells were co-incubated with the substances or vehicles for 24 h. Afterwards, the cells were analyzed for metabolic activity (**A**), HO-1 (**B**), and LC3A/B-I/II (**C**) expression as well as caspase-3 cleavage (**D**). The protein expression values were normalized to β-actin. The same actin blot was used as control in (**B**–**D**), since the proteins analyzed in these Western blots were resolved on the same gel. The percentage control provides a comparison with the vehicle-treated group (set to 100%). The data represent mean values ± SEM of *n* = 9 (**A**), *n* = 10 (**B**,**C**), and *n* = 5 (**D**) different donors. ** *p* < 0.01, *** *p* < 0.001 vs. vehicle control; # *p* < 0.05, ## *p* < 0.01 vs. CBD; one-way ANOVA with Bonferroni’s post hoc test.

**Figure 6:**
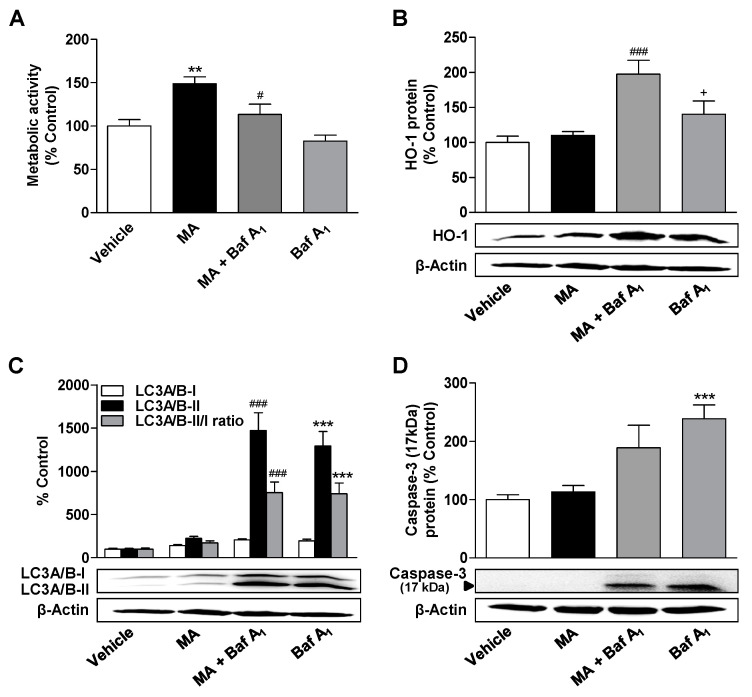
Influence of the autophagy inhibitor bafilomycin A_1_ (Baf A_1_) on the effects of MA on metabolic activity, autophagy, apoptosis, and HO-1 expression. ADMSCs were pretreated for 30 min with bafilomycin A_1_ (100 nM) and after subsequent addition of MA (3 µM) or vehicle, the cells were co-incubated with the substances or vehicles for 24 h. Afterwards, the cells were analyzed for metabolic activity (**A**), HO-1 (**B**), and LC3A/B-I/II (**C**) expression as well as caspase-3 cleavage (**D**). The protein expression values were normalized to β-actin. The same actin blot was used as control in (**B**,**C**), since the proteins analyzed in these Western blots were resolved on the same gel. The percentage control provides a comparison with the vehicle-treated group (set to 100%). The data represent mean values ± SEM of *n* = 9 (**A**,**D**) or *n* = 12 (**B**,**C**) different donors. ** *p* < 0.01, *** *p* < 0.001 vs. vehicle control; # *p* < 0.05, ### *p* < 0.001 vs. MA; + *p* < 0.05 vs. MA + Baf A_1_; one-way ANOVA with Bonferroni’s post hoc test.

Further experiments were performed with bafilomycin A_1_, a late-phase inhibitor of autophagy, to characterize the role of autophagy in processes modulated by cannabinoids. The inhibition of autophagic flow by bafilomycin A_1_ led to a significant decrease in metabolic activity increased by 3 µM CBD or MA, but not in basal metabolic activity of vehicle-treated cells (Figure 5A and Figure 6A).

In addition, an increase in cleaved caspase-3, an established apoptosis marker, was observed when the respective cannabinoid was combined with bafilomycin A_1_ (Figure 5D and Figure 6D), with bafilomycin A_1_ per se leading to increased levels of cleaved caspase-3 (Figure 5D and Figure 6D). While bafilomycin A_1_ did not affect HO-1 expression by CBD (Figure 5B), MA significantly induced HO-1 in the presence of bafilomycin A_1_ (Figure 6B). Bafilomycin A_1_ (Figure 5C and Figure 6C) alone or in combination with CBD (Figure 5C) or MA (Figure 6C) led to a significant increase in LC3A/B-II protein levels due to inhibition of the fusion of autophagosomes with lysosomes, which normally stimulates the degradation of LC3-II.

### 3.6. Inhibition of HO-1 Significantly Suppresses Apoptosis of ADMSCs Treated with Cannabinoids and the Autophagy Inhibitor Bafilomycin A_1_

**Figure 7:**
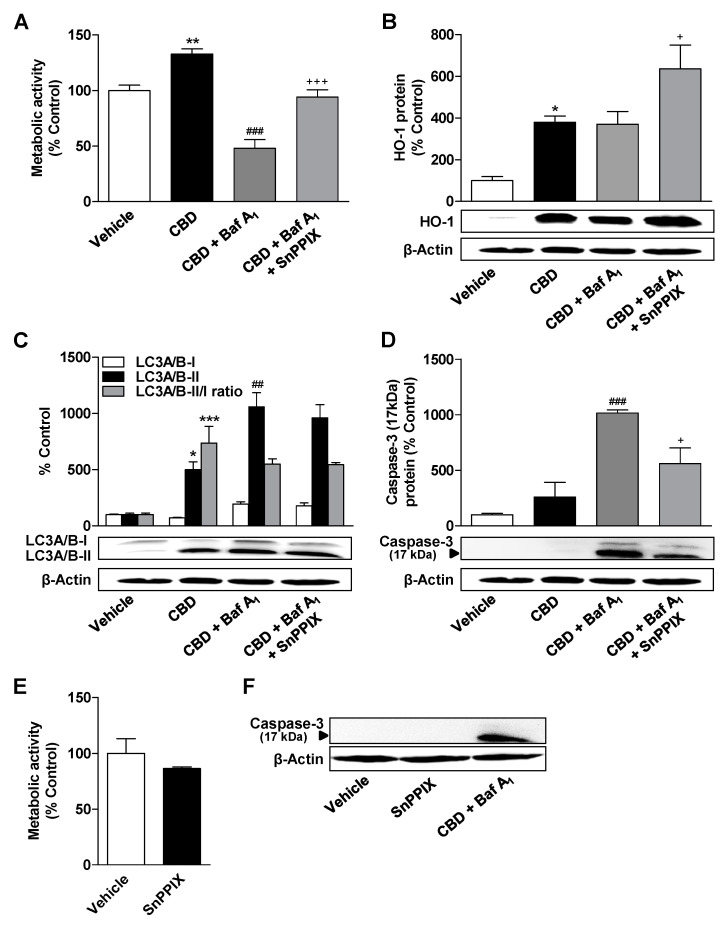
Effects of HO-1 inhibition on bafilomycin A_1_-induced changes in metabolic activity, HO-1 expression, autophagy, and apoptosis of CBD-treated ADMSCs. ADMSCs were pretreated for 30 min with the autophagy inhibitor bafilomycin A_1_ (Baf A_1_; 100 nM) and the HO-1 inhibitor SnPPIX (25 µM), and after subsequent addition of CBD (3 µM) or vehicle, the cells were co-incubated with the substances or vehicles for 24 h. Subsequently, the cells were analyzed for their metabolic activity (**A**), the expression of HO-1 (**B**) and LC3A/B-I/II (**C**), and the cleavage of caspase-3 (**D**). The effects of SnPPIX alone are shown in panel (**E**) for metabolic activity and in panel (**F**) for caspase-3 cleavage in the same experimental setting. The protein expression values were normalized to β-actin. The same actin blot was used as control in (**B**,**C**) and (**D**), since the proteins analyzed in these Western blots were resolved on the same gel. The percentage control provides a comparison with the vehicle-treated group (set to 100%). The data represent mean values ± SEM of *n* = 4 (**A**–**D**) and *n* = 6 (**E**) from different donors. * *p* < 0.05, ** *p* < 0.01, *** *p* < 0.001 vs. vehicle control; ## *p* < 0.01, ### *p* < 0.001 vs. CBD; + *p* < 0.05, +++ *p* < 0.001 vs. CBD + Baf A_1_; one-way ANOVA with Bonferroni’s post hoc test.

The functional significance of the induction of HO-1 by cannabinoids under conditions of an autophagy deficit induced by bafilomycin A_1_ was investigated with the HO-1 inhibitor SnPPIX. According to Figure 7A,D, SnPPIX led to a significant inhibition of the metabolic activity lowering and the proapoptotic effect of the bafilomycin A_1_/CBD combination, suggesting that HO-1 mediates these effects at least partially under conditions of autophagous flow inhibition. As before, bafilomycin A_1_ in combination with CBD caused a significant increase in LC3A/B-II levels (Figure 7C). An off-target effect of SnPPIX was excluded by incubating ADMSCs with SnPPIX alone, which was not associated with significant changes in metabolic viability (Figure 7E) and caspase-3 cleavage (Figure 7F). The combination of CBD and bafilomycin A_1_ is shown in Figure 7F as a positive control of caspase-3 cleavage.

**Figure 8:**
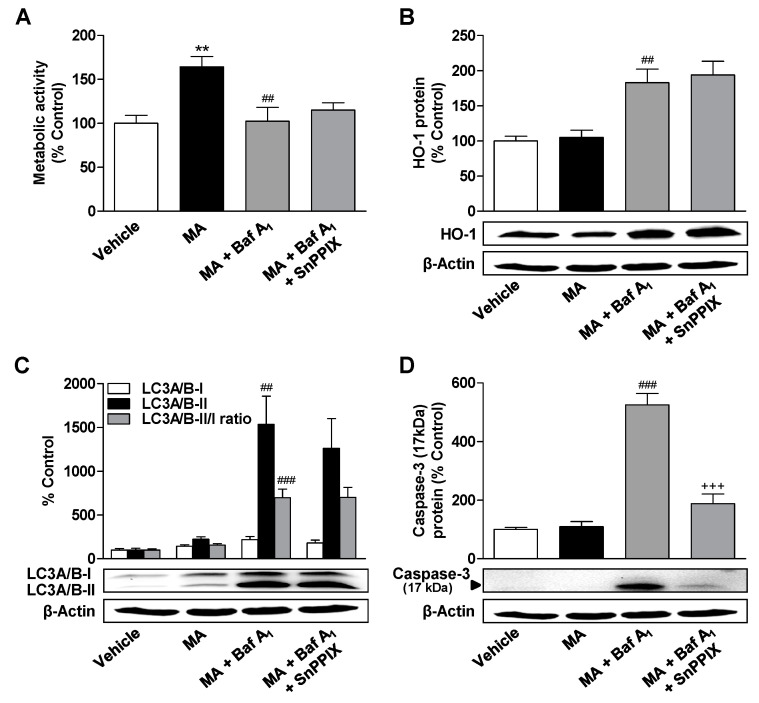
Effects of HO-1 inhibition on bafilomycin A_1_-induced changes in metabolic activity, HO-1 expression, autophagy, and apoptosis of MA-treated ADMSCs. ADMSCs were pretreated for 30 min with the autophagy inhibitor bafilomycin A_1_ (Baf A_1_; 100 nM) and the HO-1 inhibitor SnPPIX (25 µM), and after subsequent addition of MA (3 µM) or vehicle, the cells were co-incubated with the substances or vehicles for 24 h. Subsequently, the cells were analyzed for their metabolic activity (**A**), the expression of HO-1 (**B**) and LC3A/B-I/II (**C**), and the cleavage of caspase-3 (**D**). The protein expression values were normalized to β-actin. The same actin blot was used as control in (**B**–**D**), since the proteins analyzed in these Western blots were resolved on the same gel. The percentage control provides a comparison with the vehicle-treated group (set to 100%). The data represent mean values ± SEM of *n* = 6 (**A**) or *n* = 5 (**B**–**D**) different donors. ** *p* < 0.01 vs. vehicle control; ## *p* < 0.01, ### *p* < 0.001 vs. MA; +++ *p* < 0.001 vs. MA + Baf A_1_; one-way ANOVA with Bonferroni’s post hoc test.

Experiments with the same setup were also carried out with MA. Here, the presence of SnPPIX only resulted in a small, non-significant reduction of the metabolic activity loss due to bafilomycin A_1_/MA (Figure 8A). On the other hand, a highly significant inhibitory effect of SnPPIX could be demonstrated at the level of cleaved caspase-3 expression (Figure 8D). This also confirms in the case of MA the induction of a harmful proapoptotic HO-1 under conditions of autophagic flux inhibition.

## 4. Discussion

Recent studies have shown that the induction of HO-1 plays a key role in the survival of stem cells during the transplantation of adult stem cells [33]. In addition, several reports have confirmed a protective function of autophagy in stem cells [34] and a high degree of constitutive autophagy in human MSCs [35]. In this context, the present in vitro study shows that the non-psychoactive phytocannabinoid CBD and the endocannabinoid analogue MA mediate the induction of a protective autophagy and, in the case of CBD, the antioxidant enzyme HO-1 under serum-free conditions in ADMSCs.

Experimental approaches with antagonists targeting CB_1_, CB_2_, and TRPV1 showed no effect on both the bafilomycin A_1_-sensitive and thus autophagy dependently increased metabolic activity of CBD- and MA-treated ADMSCs as well as on CBD-induced HO-1 expression. Thus, both cellular responses seem to be caused by cannabinoid receptor-independent processes. In addition, a possible antagonistic effect of CBD on GPR55 as the basis of these processes was excluded by using the GPR55 agonist O-1602. For CBD, this result is consistent with our observations that CBD mediated HO-1 expression in human smooth muscle cells [36] and human umbilical vein endothelial cells (HUVEC) [37] in a likewise receptor-independent manner. In contrast, CBD has been shown to mediate its promigratory effect on ADMSCs via CB_2_ and GPR55 [12]. In the case of MA, a CB_1_ receptor-dependent autophagy was found in blastocytes [38]. In analogy to the results with CBD [12], CB_2_ receptor-dependent stimulation of ADMSC migration was also proven to be induced by inhibitors of the endocannabinoid-degrading fatty acid amidohydrolase and by the endocannabinoids anandamide and 2-arachidonoylglycerol [13]. Collectively, the investigated cannabinoids seem to mediate their biological effects via both receptor-dependent and -independent pathways dependent on cell type and/or functional effect.

There is meanwhile a large body of evidence suggesting complex crosstalks between autophagy and other stress routes such as HO-1 induction [39]. For this reason, the possible relationship between the induction of HO-1 by CBD and its simultaneous proautophagic action was investigated by use of the HO inhibitor SnPPIX or selective HO-1 siRNA. However, neither SnPPIX nor HO-1 siRNA led to a reduction of CBD-induced autophagy in ADMSCs. The role of HO-1 in the process of autophagy is controversially discussed in the literature depending on the investigated cell type. Studies on macrophages and endothelial cells showed a critical role of HO-1 in the regulation of autophagy, which was associated with pro-survival effects [40,41,42,43]. In this context, we could recently also demonstrate HO-1-dependent CBD-mediated autophagy of HUVEC [37]. On the other hand, the protective autophagy against lipotoxicity in hepatocytes mediated by the HO-1 inductor tert-butylhydroquinone proved to be HO-1-independent [44]. In addition, inhibition of the HO-1 transcription factor Nrf2 in the non-small-cell lung cancer cell line A549 was associated with a decrease in cell viability independent of autophagy. Finally, an autophagy-independent protective effect of HO-1 was described in the case of curcumin, which prevents hydrogen peroxide-induced cell death in wild-type and HO-2 knockout ADMSCs by HO-1 induction [19].

While the results of this study discussed so far have shown that autophagy caused by cannabinoids is mediated independently of HO-1, other mechanisms seem to be triggered when cellular autophagy is suppressed in the same cellular system. Accordingly, inhibition of autophagy by bafilomycin A_1_ led to significant HO-1 expression by MA and HO-1-dependent apoptosis of cannabinoid-treated ADMSCs. This is consistent with observations in different types of lung cancer cells where autophagy inhibition caused an increase in cadmium-induced HO-1 and apoptosis, while HO-1 inhibition decreased apoptosis [45]. In addition, it has recently been reported that activation of autophagy prior to hypoxia and serum withdrawal protected MSCs, while inhibition of autophagy promoted apoptosis of MSCs under these conditions [46,47]. Moreover, inhibition of autophagy led to increased glucose/reactive oxygen species (ROS)-mediated apoptosis of ADMSCs [48].

In our hands, the inhibitory effect of SnPPIX on apoptosis in cannabinoid-treated autophagy-deficient cells can be regarded as evidence that HO-1 mediates an apoptosis-enhancing effect under these conditions. Interestingly, SnPPIX showed this effect in the case of CBD-treated cells, while at the same time significantly increasing the HO-1 expression caused by the combination of CBD and bafilomycin A_1_. However, induction of HO-1 expression is a well-known effect of SnPPIX which was previously interpreted as allowing the HO inhibitor to inhibit the activity of both the preformed and the newly synthesized enzyme [49,50]. On the other hand, it should be noted that after proteolytic cleavage, HO-1, present in an enzymatically inactive form, is subject to nuclear translocation. In this context, nuclear HO-1 has been reported to be involved in increasing the proliferation and invasion of cancer cells [51] and in bortezomib-mediated chemoresistance [52]. At the mechanistic level, an activation of Nrf2 and thus the upregulation of antioxidative defenses has been described for nuclear HO-1 [53]. In our experimental system, however, the lack of effect of SnPPIX on CBD-induced increase in metabolic activity and autophagy was confirmed by experiments with HO-1 siRNA, which makes a crucial role of SnPPIX-induced enzymatically inactive HO-1 within these processes very unlikely.

The relationship between Nrf2/HO-1 and autophagy has been extensively studied since crosstalk between these two pathways was first reported in 2010, when the association between the autophagic adapter p62/SQSTM1 and Kelch-like-ECH-associated protein 1 (Keap1) was identified [54]. Since then, many mechanistic details of the Nrf2/HO-1 autophagy axis and the pathological consequences of prolonged Nrf2/HO-1 activation as a result of autophagic dysregulation, such as tissue damage and cancer, have been uncovered [55]. Inhibition of autophagy leads to accumulation of p62/SQSTM1, which binds Keap1 through the Keap1 interaction region. Thus, p62/SQSTM1 can bind and sequester Keap1 by blocking Keap1-Nrf2 binding and allowing Nrf2 to switch to the nucleus, leading to the activation of Nrf2-dependent genes such as the HO-1 gene [56]. For example, autophagy inhibition promotes epithelial mesenchymal transition through the ROS/HO-1 pathway into ovarian cancer cells, leading to migration and invasion of cancer cells [57]. In contrast, autophagy deficiency also mediated activation of the Nrf2/HO-1 pathway with protective effects by reducing damage to intestinal epithelial cells caused by indomethacin [58].

Two mechanisms for HO-1-mediated apoptosis in autophagy deficiency in ADMSCs are conceivable. On the one hand, a high degree of HO-1 expression produces a high carbon monoxide (CO) concentration and inhibits the respiratory chain at the level of cytochrome oxidase [59]. In line with this, the increased mitochondrial localization of HO-1 induced inhibition of cytochrome c oxidase activity and resulted in higher production of ROS [60]. On the other hand, sustained activation of the HO-1 transcription factor Nrf2 promotes reductive stress [61]. Our results in endothelial cells support the hypothesis of an apoptosis-inducing effect of HO-1 at a high expression rate, while a lower expression rate promotes autophagy-mediated survival [37]. A dual role of HO-1 has also been demonstrated in the nervous system [62]. Here HO-1 primarily promotes the survival of neuronal cells but induces neurodegeneration at high expression levels [62]. By use of the CRISPR/dCas9 technique, HO-1 has recently been upregulated in stem cells to an optimal extent to improve cell survival after transplantation into the ischemic heart. The HO-1 degree of expression achieved was sufficient to protect the cells and did not trigger any cytotoxic reactions [63].

Further research is also needed to understand the exact mechanism by which CBD and MA increase the metabolic activity of ADMSCs under serum withdrawal. However, it is noteworthy that CBD and MA did not cause any significant change in the number of living and dead cells in the time frame investigated, suggesting that the increased metabolic activity registered in the presence of both cannabinoids is not due to an increase in cell proliferation. Thereby, enhanced metabolic activity was measured by an increased cleavage of the stable tetrazolium salt WST-1, which mainly takes place on the cell surface [64] and is indicative of an increased glycolytic production of NAD(P)H in living cells. In this sense, cannabinoid-induced autophagy appears to be an adaptive response to increased metabolic demand, so that under these circumstances substrates for the tricarboxylic acid (TCA) cycle are preferentially provided to maintain mitochondrial energy metabolism [65,66]. In line with this, the increased metabolic activity caused by both cannabinoids was significantly reduced by the autophagy inhibitor bafilomycin A_1_, in the absence of a significant intrinsic effect of this compound on metabolic activity. With regard to possible direct mitochondrial targets of cannabinoids, previous work has also shown that the neuroprotective effect of CBD against ischaemia/reperfusion damage is associated with an increase in the activity of glucose-6-phosphate dehydrogenase, a rate-limiting enzyme of the pentose-phosphate pathway, and maintenance of the NADPH/NADP+ ratio [67]. In another study, CBD showed an inhibitory effect on the doxorubicin-induced reduced expression of markers of mitochondrial biogenesis [68].

In summary, the regulation of HO-1 expression and autophagy as well as its interplay obviously varies depending on cell type and inducer. In ADMSCs, HO-1-independent autophagy induced by cannabinoids appears to be primarily protective. A protective function could also be attributed to the autophagy induction in endothelial cells recently described for CBD. Here, the inhibition of HO-1-dependent CBD-induced autophagy led to apoptosis induction by a per se viability-promoting CBD concentration (6 µM) or to superinduction of apoptosis by a per se cytotoxic CBD concentration (10 µM) [37]. Finally, our results in vascular smooth muscle cells demonstrated a CBD-induced HO-1 expression without a concomitant proapoptotic response [36]. In cancer cells, the role of HO-1 is not yet fully understood. In two exemplary studies cited here, it was shown that HO-1 induction mediates the chemoresistance of breast cancer cells by promoting autophagy [69,70].

## 5. Conclusions

**Figure 9:**
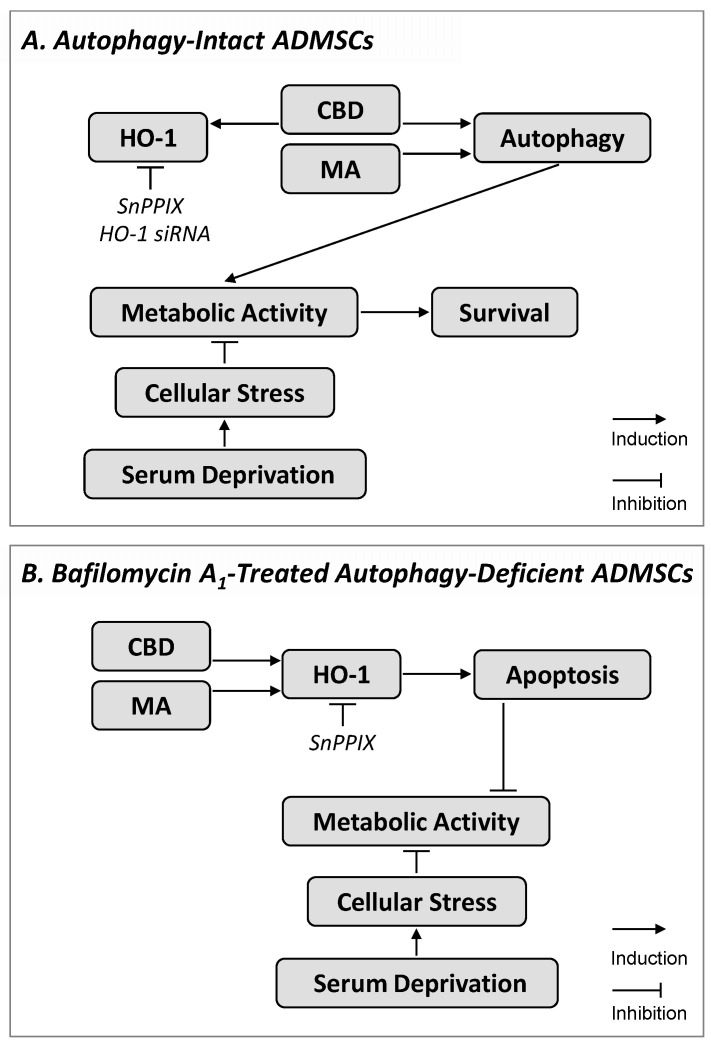
Proposed mechanism and functional consequence underlying cannabidiol (CBD)- and R(+)-methanandamide (MA)-induced HO-1 expression in ADMSCs. In cells cultured under serum deprivation (**A**) and thus exposed to time-dependent decreasing metabolic activity, CBD and MA promoted autophagy, which led to increased metabolic activity that could promote cell survival. In addition, the CBD caused an upregulation of HO-1, which, however, was not involved in increasing metabolic activity and autophagy. The involvement of different cannabinoid-binding receptors in cannabinoid effects on metabolic activity and HO-1 expression was excluded. On the other hand, inhibition of autophagy by bafilomycin A_1_ (**B**) led to apoptosis induction and inhibition of the increased metabolic activity caused by cannabinoids. Under these conditions, MA also triggered a profound HO-1 expression. Additional HO-1 inhibition finally led to a significant reversal of the bafilomycin A_1_/cannabinoid-induced apoptosis. In summary, CBD and MA protected ADMSCs under conditions of serum deprivation via HO-1-independent induction of autophagy (**A**), whereas cannabinoid-induced HO-1 contributed to apoptosis under conditions of additional autophagy inhibition (**B**). The sequence shown with inductive and inhibitory arrows connecting the boxes corresponds to the effect of CBD or MA in the absence of HO-1 inhibition. The HO-1-inhibiting strategies (SnPPIX, HO-1 siRNA) are shown in italics, whereby their target, but not their consecutive effect, is shown.

Collectively, primary ADMSCs cultivated under serum-free and thus stressful conditions develop a protective autophagy in the presence of cannabinoids (Figure 9). Although a critical role of HO-1 as a possible inductor of autophagy could be excluded, our results show induction of HO-1-dependent apoptosis in cannabinoid-treated cells when autophagy is inhibited, implying a hitherto unknown crosstalk between both systems in ADMSCs. The elucidation of the mechanisms of ADMSC protection and survival is of great importance for the therapeutic application of these cells.

## Figures and Tables

**Table 1:** Involvement of cannabinoid-binding receptors in the changes in metabolic activity and HO expression triggered by the cannabinoids (CB) studied. The cells were pre-treated for 1 h with AM-251 (CB_1_ antagonist), AM-630 (CB_2_ antagonist), or capsazepine (Capsa; TRPV1 antagonist) at 1 µM concentrations before the examined CB (each at a 3 µM concentration) or vehicle was added. Subsequently, the incubation was continued for 24 h. Afterwards, the cells were evaluated with respect to their metabolic activity by WST-1 assay. In the case of CBD experiments, the expression of HO-1 and HO-2 was additionally analyzed. Protein expression values were normalized to β-actin. The percentage control represents a comparison with the cells treated with vehicle (set to 100%) in the absence of test substance. The data represent mean values ± SEM of *n* = 4 (CBD) or *n* = 3 (MA) different donors. * *p* < 0.05 vs. vehicle control; one-way ANOVA with Bonferroni’s post hoc test. A significant effect of antagonists on basal metabolic activity and HO expression (in the absence of the respective cannabinoid) was excluded by one-way ANOVA with Dunnett´s post hoc test.

Treatment Groups	CB Corresponds to CBD (3 µM)			CB Corresponds to MA (3 µM)
	Metabolic Activity (%)	HO-1 Protein (%)	HO-2 Protein (%)	Metabolic Activity (%)
Vehicle	100 ± 35	100 ± 9	100 ± 4	100 ± 8
CB	202 ± 50	515 ± 30 *	109 ± 8	205 ± 23 *
CB + AM-630	205 ± 40	624 ± 108	102 ± 3	214 ± 26
CB + AM-251	242 ± 83	488 ± 75	89 ± 9	237 ± 29
CB + AM-630 + AM-251	220 ± 83	499 ± 91	89 ± 10	217 ± 23
CB + Capsa	211 ± 59	615 ± 122	89 ± 8	242 ± 18
Vehicle	100 ± 35	100 ± 18	100 ± 6	100 ± 26
AM-630	88 ± 6	127 ± 19	125 ± 9	87 ± 4
AM-251	125 ± 15	216 ± 75	123 ± 6	110 ± 9
AM-630 + AM-251	144 ± 19	218 ± 88	126 ± 19	120 ± 3
Capsa	93 ± 3	124 ± 13	122 ± 18	108 ± 5

**Table 2:** Involvement of GPR55 in the changes in metabolic activity and HO-1 expression triggered by CBD. The cells were pre-treated for 1 h with O-1602 at 1 µM before CBD (3 µM) or vehicle was added. Subsequently, the incubation was continued for 24 h. Afterwards, the cells were evaluated with respect to their metabolic activity by WST-1 assay or for the expression of HO-1 by Western blot. Protein expression values were normalized to β-actin. The percentage control represents a comparison with the cells treated with vehicle (set to 100%) in the absence of test substance. The data represent mean values ± SEM of *n* = 3 different donors. Statistical significances were excluded by one-way ANOVA with Bonferroni´s post hoc test.

Treatment Groups	Metabolic Activity (%)	HO-1 Protein (%)
Vehicle	100 ± 29	100 ± 16
3 µM CBD	174 ± 32	277 ± 64
CBD + O-1602	188 ± 33	255 ± 61
O-1602	106 ± 10	103 ± 21
